# Natural history of gastroesophageal reflux in infancy: new data from a prospective cohort

**DOI:** 10.1186/s12887-020-02047-3

**Published:** 2020-04-07

**Authors:** Marlène Curien-Chotard, Prévost Jantchou

**Affiliations:** 1 Pediatric Unit, Besançon Teaching Hospital , 3 Boulevard Alexandre Fleming, 25000 Besançon, France; 2 CHU Sainte-Justine. 3175 Côte Sainte Catherine, H3T IC5, Montréal, Québec Canada; 3 Université de Montreal, Montreal, Canada

**Keywords:** Gastroesophageal reflux, Gastroesophageal reflux disease, Infancy, Infant, Infant Gastroesophageal Reflux Questionnaire Revised, I-GERQ-R, pediatric practice, regurgitation

## Abstract

**Background:**

Gastroesophageal reflux (GER) is common in infants. Gastroesophageal reflux disease (GERD) is defined as GER leading to troublesome symptoms that affect daily functioning and/or complications. This study is aimed at determining the prevalence and progression of GER and GERD in a cohort of healthy term infants from birth to 12 months old.

**Methods:**

We conducted a prospective cohort study including all full-term living neonates born at Besançon Teaching Hospital, France. Parents completed a clinical report form and the Infant Gastroesophageal Reflux Questionnaire-Revised (I-GERQ-R) at 1, 3, 6, 10, and 12 months of age. GER was defined as score ≥ 1 to the first question with I-GERQ-R score < 16, and GERD as score ≥ 1 to the first question with I-GERQ-R score ≥ 16. Regurgitation was based on the answer to the first question of the I-GERQ-R as anything coming out of the mouth daily.

**Results:**

157/347 births were included (83 boys). The prevalence of regurgitation at least once a day was 45.7% overall. In total: 72, 69, 56, 18, and 13% of infants regurgitated at least once a day at 1, 3, 6, 10, and 12 months of age, respectively. Physiological GER affected 53, 59, 51, 16, and 12% of infants; GERD, 19, 9, 5, 2, and 2%, respectively. Two risk factors were identified: family history of GER and exposure to passive smoking. Treatment included dietary modification (14%) and pharmacotherapy (5%).

**Conclusion:**

Physiological GER peaked at 3 months, GERD at 1 month. Most cases resolved on their own. GER and GERD are very common in the infant’s population and parents should be reassured/educated regarding symptoms, warning signs, and generally favorable prognosis. I-GERQ-R is useful to the clinical screening and follow up for GER and GERD.

## Background

Gastroesophageal reflux (GER) is a common and normal physiological process in children whatever their age [1]. In 2009 both the North American Society for Pediatric Gastroenterology, Hepatology, and Nutrition (NASPGHAN) and the European Society for Paediatric Gastroenterology, Hepatology, and Nutrition (ESPGHAN) defined GER as the passage of gastric contents into the esophagus with or without regurgitation and vomiting [1]. GER is usually the consequence of an insufficiency of the lower esophageal sphincter tonus and other anatomical factors [2], GER can occur after meals several times a day, with little or no symptoms [1]. The 2018 reflux guidelines used the same definition for GER and GERD as the 2009 guidelines [3].

The diagnosis of GER and GERD relies on clinical symptoms and signs (medical history and physical examination) but these are non-specific, particularly in infants [1]. Additional diagnostic investigations are needed to qualify and quantify GERD and to rule out conditions other than GERD [3].

The prevalence of GER and GERD vary according to the population, the study design (cross-sectional or longitudinal), and the diagnostic criteria (visible symptoms vs. validated questionnaire). A French study estimated that 24.4% of infants (0–23 months) had symptoms of GER and the prevalence of GERD was 12.6% [4]. GER has been reported as a common condition until 3 months of age, with a reported peak in regurgitation at 4 months of age (67% of infants). At 6 months, 23% of infants were symptomatic [5]. Another study published a peak in spitting/vomiting (“spilling”) at 3–4 months of age (41% of infants) [6]. Overall 12% of children aged 1 to 12 months were diagnosed with infant regurgitation [7]. A prospective study of healthy infants from birth to 6 months in the USA, indicated a peak prevalence of regurgitations ≥ once/day of 83% at 4 months of age and of GERD 25% at 1 month of age, decreasing thereafter [8].

During the few years, the number of parents seeking pharmacological treatment for GER in our clinical practice has been increasing with a corresponding increase in prescriptions for drugs such as proton pump inhibitors (PPIs) in infants [9–12]. However, several randomized controlled trials showed no difference between PPI and placebo on the reduction of symptoms of GERD [3].

The primary aim of our study was to determine the prevalence and the progression of GER and GERD in a cohort of infants from birth to one year of age in the eastern part of France. The secondary aims were to examine the pattern of drug prescription and to identify risk factors for GER and GERD in this cohort.

## Methods

Study design: a prospective cohort study.

### Participants

All infants born at term at Besançon Teaching Hospital, France, during the two months of study recruitment (October 2009–December 2009) were eligible for this study. The inclusion criteria were as follows: (1) Parents able to read and write French and (2) Familial residence at the same address without any plans to move for at least one year. The Exclusion criteria consisted of the following: (1) preterm birth, (2) Pierre Robin syndrome or other otolaryngological or digestive defects (diaphragmatic hernia, omphalocele, and gastroschisis), (3) congenital heart disease, (4) neonatal neurological disorder, (5) other congenital malformations and (6) an intestinal surgery in the first three months of life.

Data collection: after informed consent, each family was asked to fill the Gastroesophageal Reflux Questionnaire Revised (I-GERQ-R) alongside a case report form.

The I-GERQ was developed by Orenstein et al. [13, 14] and Kleinman et al. [15] to assess the symptoms of GER and GERD in children. The I-GERQ is a shorter version of the I-GERQ-R that was developed and validated in seven countries.

The mother or the father completed five self-reported questionnaires at home when the infant was 1, 3, 6, 10, and 12 months old. One postal mail reminder was sent to non-responders and those who still did not respond were phone called. Each questionnaire consisted of the 12 I-GERQ-R items along with additional questions. The family history of reflux was defined as a first or second degree relative with a history of heartburn, regurgitation, GER or GERD. We requested information on the age of introduction of solid food, sleep disturbances, and psychomotor development evaluated by age-appropriate developmental delay in follow-up questionnaires. Data on the different treatments for GER and GERD were also collected in each questionnaire: thickening agent, alginate (sodium alginate), histamine receptor antagonist (ranitidine), prokinetic (domperidone, cisapride) or proton pump inhibitor (omeprazole, esomeprazole). In addition, the occurrence of hospitalization during the study was self-reported by the parents and validated by medical chart reviews. Finally, parental perception of the global child’s health was assessed at each questionnaire.

Regurgitation was based on the answer to the first question of the I-GERQ-R as anything coming out of the mouth daily. A score ≥ 1 on the first item (presence of regurgitations) combined with an I-GERQ-R score < 16 indicated GER. A score ≥ 1 on the first item with an I-GERQ-R score ≥ 16 indicated GERD [15].

The hospital ethics committee approved the study and all parents signed informed consent forms. The study was also approved by the French Advisory Committee on Information Processing in Material Research in the Field of Health (agreement#2014/165).

### Statistical analysis

Numerical data were expressed as median (interquartile range) or as mean ± standard deviation according to their parametric distribution. Qualitative data were expressed as a number (percentage). We assessed the prevalence of GERD at each questionnaire by the proportion of children with a GER or GERD score according to the total number of parents who answered the questionnaires. We did not include missing data in the calculation of prevalence. The clinical factors associated with the risk of GER or GERD were analyzed using chi-squared tests in univariate analysis and a multivariate logistic regression. *P* values < 0.05 were considered statistically significant. All analyses were performed using SAS software, version 9.1 (SAS Institute, Cary, NC).

## Results

**Fig. 1:**
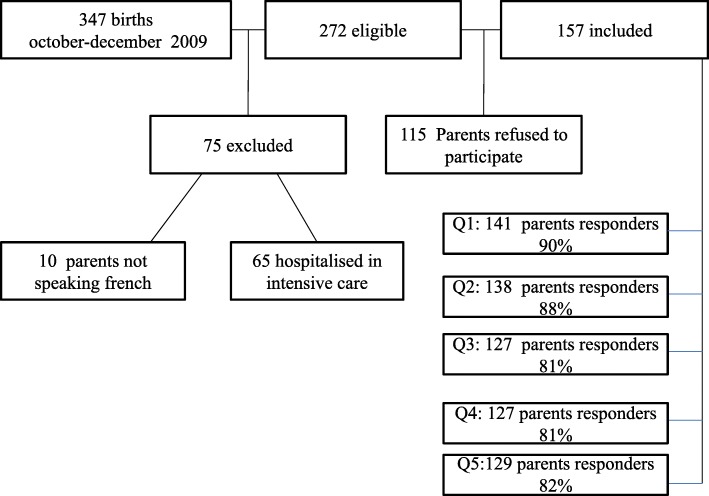
Study flowchart of patient’s selection and follow-up

Among the 347 living neonates born during the inclusion period of 2 months, we included 157 (83 boys, 53%) in the study. The cohort included two pairs of twins, one homozygous, and the other heterozygous. Prematurity (*n* = 75) was the main reason of non-eligibility. Among the eligible infants, 42% of families declined participation (Fig. 1).

In total, 90% of the participants completed the questionnaire at 1 month, 88% at 3 months, 81% at 6 months and 10 months and 82% at 12 months. Only 10 parents did not answer any of the questionnaires. They were excluded from the analysis. The median (range) age of responders was 25.2 years (20.3–43.8), vs. 28.7 years (22.4–37.4) for the non-responders. However, the number of older children was similar between the two groups.

Most of the questionnaires were completed by the mother alone (90%). The general characteristics of the study population are displayed in Table 1.

**Table 1:** General characteristics of the study population

Sex, N(%)	
Male	77 (54.6)
Female	64 (45.4)
**Maternel age (min-max)**	25.2 (20.3–43.8)
**Questionnaire Responders, N(%)**	
Mother	131 (92.9)
Father	4 (2.8)
Mother and Father	5 (3.6)
Missing data	1 (0.7)
**Family history of Gastroesophageal reflux, N(%)**	55 (39)
**Number of older children, N(%)**	
None	45 (32)
1	44 (31.2)
≥ 2	52 (36.8)
**Baseline diet, N(%)**	
Breastfeeding	69 (48.9)
Breastfeeding and infant formula	31 (22)
Infant formula	41 (29.1)
**Passive Smoking exposure, N(%)**	
Paternal smoking	31 (22)
Maternal smoking	5 (3.6)
**Body positioning during sleep, N(%)**	
Dorsal decubitus	134 (95.1)
Ventral decubitus	3 (2.1)
Lateral decubitus	3 (2.1)
Missing data	1 (0.7)
**Infant’s hospitalization at least one questionnaire, N(%)**	
Any hospitalisation	29 (20.6)
No related to GER	27 (19.1)
Related to GER	2 (1.4)
**Developmental milestones, N(%)**	
Sitting position acquired before 9 months	122 (96)
Standing position acquired before 12 months	89 (69)
**Age of Introduction of solid foods, median (min-max)**	
Fruits	6.1 (5.2–6.1)
Vegetables	6.3 (5.3–7.4)
Fish and/or meet and/or egg	6.4 (6–7.3)
Gluten	6.5 (6.1–7.3)
**GERD on at least one questionnaire, N(%)**	27 (19.1)
**Proton pump inhibitor treatment, N(%)**	6 (4.3)
**Prokinetic treatment, N(%)**	9 (6.4)

**Fig. 2:**
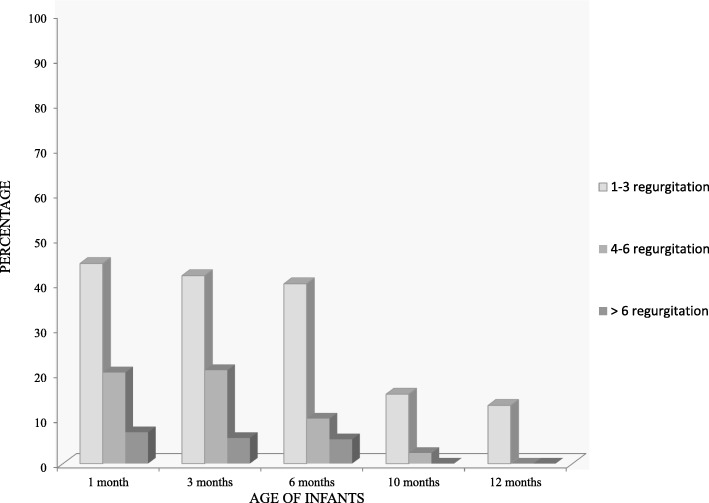
Incidence of daily regurgitation between one and twelve months of age

The regurgitation incidence during the first month of life was 72.3% (95% confidence interval (CI) [64.9–79.7]). The percentage of infants regurgitating at least once a day decreased thereafter: 69, 56, 18, and 13% at 3, 6, 10, and 12 months, respectively. Few infants regurgitated more than 6 times a day (7% at 1 month and 6% at 3 and 6% at 6 months of age, and none thereafter) (Fig. 2).

**Fig. 3:**
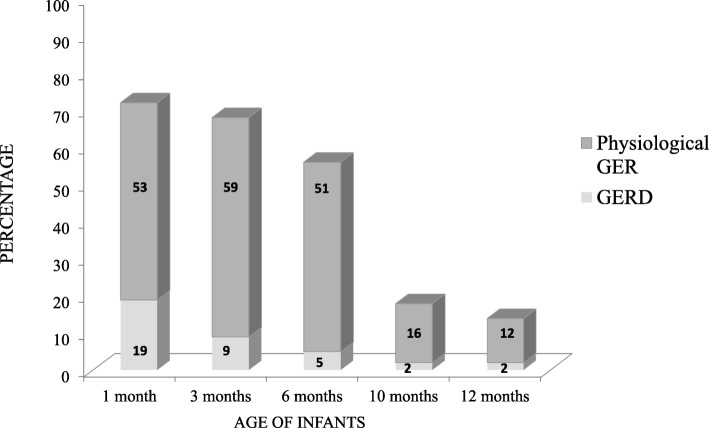
Incidence of GER and GERD between one and twelve months of age

The progression of physiological GER and GERD were markedly different. Whereas physiological GER peaked at 3 months of age (59.4%), GERD peaked at 1 month of age (19%) (Fig. 3). The prevalence of GERD thereafter dropped from 9% at 3 months to 2% at 12 months of age. We also noticed a favorable outcome for GERD: of the eleven cases with persistently elevated GERD scores (I-GERQ-R score ≥ 16 on at least 2 questionnaires), 7 resolved without treatment and 4 with a prokinetic agent and/or antacid. Furthermore, in 3 of 6 infants who presented GERD at only one time-point (4 infants at 3 months and 2 infants at 10 months), elevated scores were associated with concurrent diseases the day before completion of the questionnaire: bronchiolitis (*n* = 1), hives (n = 1), and fever (n = 1).

Two risk factors were identified for GER and GERD at 1 month of age: family history of GER (OR: 2.9–95% CI [1.2–7.1] and OR: 4.8–95% CI [1.6–14.4]- respectively) and exposure to paternal smoking (Table 2). At 3 months, the family history of GER was significantly associated with the risk of GER in the infant (−OR (95%CI): 31[3.6–265.1]-) (Table 3).

**Table 2:** Multivariate analysis of the risk factors for GER and GERD at one month of age (*N* = 141)

	No GER *N* = 39 n(%)	GER *N* = 75 n(%)	GERD *N* = 27 n(%)	***p***	OR [95% CI] GER vs No GER	OR[95% CI] GERD vs No GER
Family history of GER	8 (20.5)	32 (42.7)	15 (55.6)	0.01 *	2.9 [1.2–7.1]	4.8 [1.6–14.4]
Sex-male	19 (48.7)	43 (57.3)	15 (55.6)	0.68	1.4 [0.6–3.1]	1.3 [0.5–3.5]
Exclusive breastfeeding	13 (33.3)	42 (56.0)	14 (51.8)	0.09	2.5 [1.1–5.7]	2.1 [0.8–5.9]
Paternal smoking	15 (38.5)	21 (28.0)	15 (55.6)	0.03*	0.6 [0.2–1.4]	2.0 [0.7–5.4]
Maternal smoking	6 (15.4)	13 (17.3)	6 (22.2)	0.76	1.1 [0.4–3.3]	1.6 [0.4–5.5]

*GER* Gastroesophageal reflux

*GERD* Gastroesophageal reflux disease

* *p* < 0.05

**Table 3:** Multivariate analysis of the risk factors GER and GERD at three months of age (*N* = 138).

	No GER *N* = 43 n(%)	GER *N* = 82 n(%)	GERD N = 13 n(%)	***p***	OR [95% CI] GER vs No GER	OR [95% CI] GERD vs No GER
Family history of GER	12 (27.9)	26 (31.7)	12 (92.3)	< 0.0001*	1.2 [0.5–2.7]	31 [3.6–265.1]
Sex-male	20 (46.5)	44 (53.7)	10 (76.9)	0.16	1.33 [0.6–2.8]	3.83 [0.9–15.9]
Exclusive breastfeeding	11(25.6)	27 (32.9)	5 (38.5)	0.6	1.43 [0.6–3.3]	1.82 [0.5–6.7]
Paternal smoking	18 (41.9)	24 (29.3)	5 (38.5)	0.3	0.57 [0.3–1.2]	0.87 [0.2–3.1]
Maternal smoking	9 (20.1)	12 (14.6)	2 (15.4)	0.7	0.65 [0.2–1.7]	0.69 [0.1–3.7]

*GER* Gastroesophageal reflux

*GERD* Gastroesophageal reflux disease

* *p* < 0.05

The rate of exclusive breastfeeding was 49% at 1 month, 31% at 3 months and 9% at 10 months of age. At 1 month the rate of breastfeeding was 33.3, 56.0, and 51.8% in the No GER, GER and GERD, groups respectively (Table 3).

The main treatment of GER was dietary change: overall, during the first year of life, 14% of infants, were given a thickening agent; 5% were treated with a pharmacological agent (antacid, prokinetic, and/or PPI). The number of treated infants peaked at 3 months, with 20% of infants receiving a thickening agent. The prescription of pharmacological treatment consisted mainly of antacid, prokinetic, and PPI peaking at 10, 5, and 3% respectively, at 3 months of age. Only 40% of infants with a diagnosis of GERD based on I-GERQ-R score ≥ 16, were treated with pharmacological treatments (6/15). However, 60% of infants who did not meet diagnostic criteria for GERD, received a pharmacological treatment (9/15). Approximately 5% of the cohort received a PPI treatment at any time-point (Table 1) but only 17% of these PPI prescriptions were justified (according to the I-GERQ-R score ≥ 16) whereas 83% were unjustified (I-GERQ-R score < 16), *p* = 0.87.

The median (range) age of introduction of solid food was 6.5 (5.2–9.3) months of age. There was no significant difference regarding the timing of the introduction of solids in infants with or without GER.

The majority of parents reported sleep disturbance in the first month of life (45%) with a statistically significant difference in infants with or without GERD *(p* = 0.0028*).* Ten percent of parents were worried about their infant’s health on at least one questionnaire.

## Discussion

In this prospective cohort study, we aimed at determining the prevalence of GER and GERD in infants followed longitudinally from birth to 12 months of age. 157 infants were included of the 272 eligible. The rate of refusal to participate was 42%. This figure is comparable to other published population-based studies of pediatric GER*.* In 2002, Martin et al. approached 3200 mothers and 2000 agreed, wich suggested that 1200 mothers (37.5%) refused to participate [6]. Almost half of the infants aged less than 12 months experienced at least one daily episode of regurgitation, mainly in the first 3 months of life. The prevalence of physiological GER peaked at age 3 months, 60% of infants, while GERD peaked at age 1 month; almost 20%. The risk factors for GER and GERD were family history of GER, and exposure to passive smoking.

More than two-thirds of infants regurgitated daily at 1 month and this figure gradually declined until 12 months of age. Although the timeframes were somewhat different, the rates were similar to those published by Nelson et al. In a cross-sectional survey from pediatric practice, half of the 948 infants regurgitated at least once a day between 0 and 3 months, peaking at 67% at 4 months, and decreasing thereafter to 61% at 6 months and 21% at 7 months of age [5]. Thus, the prevalence of regurgitation remained unchanged over the years. Our results are also similar to those published elsewhere. In the first prospective longitudinal study including 4672 infants (2002), “visible” regurgitation (“spilling”) peaked at 3–4 months of age (41% of infants) and decreased to 5% at age 13–14 months [6]. In a survey of pediatricians in 2005, the regurgitations were the most common gastrointestinal “symptom” in infants aged 0–6 months, affecting 23.1% of infants [16]. The average prevalence of regurgitation over the first 2 years of life was 12% and common before 5 months of age, according to Campanozzi’s evaluation using Rome II criteria and the validated I-GERQ score [7]. Using I-GERQ, Salvatore’s performed a cross-sectional study of 200 infants from 0.5 to 12 months of age and found an average of 45% of healthy infants regurgitating daily [17]. A prospective study in 2010 of 128 infants in Michigan, USA, using an I-GERQ-R score, determined the prevalence of regurgitations at least once a day to be 82, 77, 83, 67% at 1, 2, 4, and 6 months, respectively, with GERD in 25.5, 12.5, 8.0, and 2.9% of infants, respectively [8].

In 2012, a French study included 10,394 patients, aged 0–17 years, seen by the family physicians (general practitioners and paediatricians). For patients presenting with GER symptoms, a 24-item questionnaire was completed by the physician. Martigne et al. estimated that 24.4% of infants (ages 0–23 months) had symptoms of GER. Among infants, 81% had GER symptoms at least twice a day. The high prevalence of regurgitation may be explained by the enrollment of children who were seen in the physician’s office. In addition, the diagnosis was established by the physician and not on parental reports of their children’s symptoms [4].

In the present study, environmental tobacco smoke exposure was associated with an increased risk of GER and GERD at 1 month of age. The prevalence of passive smoking was high. Overall, 39.7% of infants were exposed to passive smoking. Paternal smoking was more frequent than maternal smoking. In comparison, in Lebanon, in 2015, 48.2% of children aged between 3 and 15 years (527) were exposed to smoke at home [19]. In Macao, Zheng et al, demonstrated that 41.3% of children aged 6–14 years old (875 children) were exposed to tobacco. Among 415 smokers, the father’s smoking represented a large majority (92%) [20].

Tobacco smoke is known to induce the relaxation of the lower esophageal sphincter [18]. Two studies had already identified tobacco exposure as a risk factor for GER [18] and GERD [14], although other studies did not report that association [6, 7, 17].

Gender was not associated with an increased risk of GER or GERD, similar to what was reported in previous pediatric studies in which the occurrence of pathological pH monitoring data was equally frequent in boys and girls [21]. Likewise, in a systematic review that included 31 studies on the risk factors associated with GERD (only one pediatric study) the authors showed that sex was not associated with GERD [22].

In the current study, a family history of GER in the first or second-degree relatives was a strong risk factor for physiological GER and GERD in the first 3 months of age. Four studies reported an association between having GERD symptoms and having a genetically related family member with gastrointestinal symptoms [23–26].

We did not find a statistically significant relationship between breastfeeding and the risk of GER nor GERD, consistent with other studies showing no protective benefit of breastfeeding on GER/GERD [6, 8, 14]. Nonetheless, Heacock et al. suggested that episodes of reflux were shorter in breastfed infants [27]. In Campanozzi’s cohort, breastfeeding reduced the frequency of regurgitations as compared to formula feeding [7].

Management options for physiological GER include lifestyle changes [7] (feeding and positional modifications), parental education, reassurance and anticipatory guidance [2]. Providing parental education and support as part of the treatment of GERD in association with pharmacologic treatment [3]. The use of alginates may slightly improve visible regurgitation/vomiting as signs and symptoms of GER [3]. Severe GERD may be treated with acid suppressors especially in older infants [2, 28]. The use of PPIs as the first-line treatment of reflux-related erosive esophagitis in infants is recommended [3]. The use of histamine receptor antagonists is recommended if PPIs are contra-indicated or not available [3].

Approximately 5% of the cohort received PPIs at any time-point**.** In recent decades, the prescription of PPIs has expanded in the pediatric population [9, 11]. De Bruyne et al. demonstrated an increase in acid-suppressant prescriptions among Belgian pediatricians including PPIs between 1997 and 2009 [10]. In a retrospective analysis of PPI prescribing patterns for newborns and infants, the authors estimated that prescriptions had more than doubled from 2004 to 2008 in the United States [12]. Although PPIs are generally highly effective for treating erosive disease [29], they are not effective in reducing GERD symptoms in infants as demonstrated in a systematic review [30]. Moreover, the gastric contents of the milk-fed infants are non-acidic during a large part of the day, obviating the need for suppression of gastric acid secretion. The use of PPIs may increase the risk of adverse events such as necrotizing enterocolitis, pneumonia, upper respiratory tract infection, urinary tract infection, or *Clostridium difficile* infections [3]. In addition, PPI may increase the risk of bone fracture, dementia, myocardial infarction, renal disease [3], vitamin B12 deficiency, and hypomagnesaemia [11]. However, these adverse events are controversial in studies [3]. Of notice, in our cohort, the symptoms of GER and GERD largely resolved over time. The use of PPIs in this population is therefore questionable. Several randomized controlled trials in infants with GERD showed no difference between PPI and placebo [3].

A major strength of our study was its prospective design. Furthermore, we included a relatively long follow-up (one year), as opposed to some studies that ended after 6 months. The population was based on infants born at term and healthy, remaining without serious complications for the first 3 months of life. With an almost 90% rate of return, our study results can, therefore be generalized to any well-baby visit. In addition, comparing our results to those from an academic rural population in the USA showed little difference to our experience in a small urban community in France, thus rendering our findings extrapolative to other populations [8].

Nonetheless, there are several limitations to this study. First, we used the I-GERQ-R questionnaire as a surrogate for accurate gastroesophageal reflux diagnosis. This score was validated in many languages including French and was developed to distinguish GER from GERD on clinical criteria. However, items such as the frequency of crying and other infant behaviors can be influenced by underlying diseases and falsely increase the total score. Van Howe and Storms suggested that a high score in the first months of life may reflect colic-associated symptoms and that GERD should not be considered a likely diagnosis until 3 months of age [8]. While sensitive as a screening tool, the I-GERQ-R score should probably be complemented by other, albeit more invasive, tests to exclude other potential diseases (e.g. Eosinophilic esophagitis) in severe or persistent cases despite pharmacological treatment [3, 31]**.** The second limitation of our study was that participating mothers were somewhat younger than non-participants mothers. Nonetheless, the number of older children was the same between the two groups. Third, we did not ask questions to evaluate the socioeconomic status. This potential selection bias may have affected risk factors like environmental tobacco smoke exposure. Lastly, the sample size was insufficient to investigate factors associated with the persistence of GERD at older ages where the prevalence of GERD was low.

## Conclusion

In conclusion, in this prospective cohort study, the prevalence of GER was high in infants aged less than one year but most cases resolved spontaneously with time as infants got older. We identified two risk factors, one of which is preventable: environmental tobacco smoke exposure and family history of GER. I-GERQ-R is useful for clinical screening and follow up of GER and GERD.

## Data Availability

The datasets used and/or analyzed during the current study are available from the corresponding author on reasonable request.

## Abbreviations

GER: Gastroesophageal reflux
GERD: Gastroesophageal reflux disease
I-GERQ-R: Infant gastroesophageal reflux questionnaire revised
PPI: Proton pump inhibitor
